# Supplementary material for: A Novel lncRNA SAAL Suppresses IAV Replication by Promoting Innate Responses
Source: Microorganisms. 2022 Nov 25;10(12):2336. doi: 10.3390/microorganisms10122336 (PMC9785332; doi:10.3390/microorganisms10122336)
Supplement: Supplementary file 1 [file microorganisms-10-02336-s001.zip › microorganisms-2034557-supplementary.pdf]

Table S1. Primers used in qRT-PCR.

Primer	Sequence (5′ → 3′)	Targeting genes
TCONS_00218279-F	AAAATGTTGTCTTCTCCCCAT	TCONS_00218279
TCONS_00218279-R	TCTGTGAGGTTGAACTTGAGA	
TCONS_00237522-F	ATGTGCTAATATGTACAATGA	TCONS_00237522
TCONS_00237522-R	GACCAAAATATAAACAGAGAA	
TCONS_00296065-F	CTTCTTCAGATAGGTTACTTG	TCONS_00296065
TCONS_00296065-R	AAATTTGAAACCAAGAAGAAA	
TCONS_00404240-F	AGCTATTGATTGCCTCATCCT	TCONS_00404240
TCONS_00404240-R	ATGTCATGTACCCTAGTGTCT	
TCONS_00748040-F	CCAGAAGGATAGCTTAGTGAG	TCONS_00748040
TCONS_00748040-R	TGGGACACGTGGAAGTCAGAG	
TCONS_00778347-F	AGGTAATGAATGAGATAGGA	TCONS_00778347
TCONS_00778347-R	TACAAGTAAAGCCTCCATAA	
NONMMUT000378.2-F	GTTTTATCCAACTCATCCCT	NONMMUT000378.2
NONMMUT000378.2-R	AATAATGGGAATCAGTGAAC	
NONMMUT058145.2-F	AGTCTTCCACTAGCAGCCTT	NONMMUT058145.2
NONMMUT058145.2-R	TGGCTTACAGGTTCAGAGGT	
TCONS_00651694-F	TGATGCCATAAAGTGCTTGT	TCONS_00651694
TCONS_00651694-R	CTTTACTTTGGGGTCTCTGT	
TCONS_00974383-F	CCAAACTCATTCTCTGACTC	TCONS_00974383
TCONS_00974383-R	TTCCAGGATTCAATTTGAGT	
TCONS_00424733-F	CAAGTCCCTACCTCCTCCCT	TCONS_00424733
TCONS_00424733-R	AAGAAGCCCCATACAGAATA	
NONMMUT121027.1-F	ATATTAGCTATCTATCGGAT	NONMMUT121027.1
NONMMUT121027.1-R	GATCAATGGCTTATGCTCTA	
NONMMUT074110.2-F	GGGTTTGGATGAATACAGAT	NONMMUT074110.2
NONMMUT074110.2-R	CAATGACCTTGTTCTTAACT	
NONMMUT145028.1-F	CACTCGTAAACCACAGATCT	NONMMUT145028.1
NONMMUT145028.1-R	TATCAACAAAACCCATAACA	
NONMMUT111155.1-F	GCTGGCATTTGTGTTCTCTT	NONMMUT111155.1
NONMMUT111155.1-R	ATCAAGTAACATATAAAGGC	
TCONS_00373113-F	TGATGTAGCACTTGATTTAA	TCONS_00373113
TCONS_00373113-R	CATCTTACAAATGAGAAATT	
TCONS_00938890-F	TAACTCCTGATCTCAAGAAC	TCONS_00938890
TCONS_00938890-R	CAAGAATGTGTTAGAGCCAA	
NONMMUT032524.2-F	GAAAACGAATGAGGGTTAGAAGT	NONMMUT032524.2
NONMMUT032524.2-R	GGCGGAGATCCCTCTGTACTTTG	
NONMMUT061473.2-F	ATGCAACGGTACCAAAGAAGCTC	NONMMUT061473.2
NONMMUT061473.2-R	ATCCGGAGTTGAACACATTCCTT	
NONMMUT127241.1-F	TGAGTGTAGCTGGATAGACAAGA	NONMMUT127241.1
NONMMUT127241.1-R	CAAATTCACTTACAGAGTGCTGA	
